# Internal Gene Cassette from a Genotype S H9N2 Avian Influenza Virus Attenuates the Pathogenicity of H5 Viruses in Chickens and Mice

**DOI:** 10.3389/fmicb.2017.01978

**Published:** 2017-10-11

**Authors:** Xiaoli Hao, Jiongjiong Wang, Jiao Hu, Xiaolong Lu, Zhao Gao, Dong Liu, Juan Li, Xiaoquan Wang, Min Gu, Zenglei Hu, Xiaowen Liu, Shunlin Hu, Xiulong Xu, Daxin Peng, Xinan Jiao, Xiufan Liu

**Affiliations:** ^1^Animal Infectious Disease Laboratory, School of Veterinary Medicine, Yangzhou University, Yangzhou, China; ^2^Jiangsu Co-innovation Center for Prevention and Control of Important Animal Infectious Diseases and Zoonosis, Yangzhou University, Yangzhou, China; ^3^Key Laboratory of Prevention and Control of Biological Hazard Factors (Animal Origin) for Agri-food Safety and Quality, Ministry of Agriculture of China, Yangzhou University, Yangzhou, China; ^4^Jiangsu Key Laboratory of Zoonosis, Yangzhou University, Yangzhou, China

**Keywords:** avian influenza, H9N2, H5, internal gene cassette, genotype S

## Abstract

H9N2 avian influenza virus (AIV) of genotype S frequently donate internal genes to facilitate the generation of novel reassortants such as H7N9, H10N8, H5N2 and H5N6 AIVs, posing an enormous threat to both human health and poultry industry. However, the pathogenicity and transmission of reassortant H5 viruses with internal gene cassette of genotype S H9N2-origin in chickens and mice remain unknown. In this study, four H5 reassortants carrying the HA and NA genes from different clades of H5 viruses and the remaining internal genes from an H9N2 virus of the predominant genotype S were generated by reverse genetics. We found that all four H5 reassortant viruses showed attenuated virulence in both chickens and mice, thus leading to increased the mean death times compared to the corresponding parental viruses. Consistently, the polymerase activity and replication ability in mammalian and avian cells, and the cytokine responses in the lungs of chickens and mice were also decreased when compared to their respective parental viruses. Moreover, these reassortants transmitted from birds to birds by direct contact but not by an airborne route. Our data indicate that the internal genes as a whole cassette from genotype S H9N2 viruses play important roles in reducing the pathogenicity of the H5 recombinants in chickens and mice, and might contribute to the circulation in avian or mammalian hosts.

## Introduction

Avian H5 and H9N2 influenza viruses are two major subtypes that circulate in poultry populations globally (Su S. et al., 2015). In 1996, the Asian lineage of the highly pathogenic H5N1 AIV was first detected from a farmed goose in China (Xu et al., 1999). In 1997, this subtype virus was found to be capable of transmitting from birds to humans in Hong Kong (Claas et al., 1998). Since 2003, the H5N1 virus has become a threat to both public health and poultry industry worldwide. Currently, the HA gene of the H5 viruses has evolved into 10 clades, some of which further diverged to different orders of subclades (Smith et al., 2015). Among them, a new subclade 2.3.4.4 with different NAs, such as H5N2, H5N3, H5N5, H5N6 and H5N8 (H5NX) has emerged and spread in recent years, raising a great concern in Asia, Europe, Africa and North America (WHO, 2015). In particular, H5N6 has superseded H5N1 for the predominant subtypes in southern China (Bi et al., 2016). Although H5 subtype has not been reported to sustain human-to-human transmission, the virus still remains a potential pandemic threat via mutations or reassortment with other influenza A viruses (IAVs) (Imai et al., 2012; Zhang et al., 2013; Hao et al., 2016).

Similarly, the low pathogenic avian influenza virus (LPAIV) of H9N2 subtype has evolved into more than 102 genotypes and become endemic in most poultry raising areas since its first emergence in chickens in 1994 in China (Su S. et al., 2015). Among these genotypes, the genotype S was first identified in chickens in eastern China in 2007. We previously demonstrated that H9N2 viruses of genotype S have become predominant in chickens since 2010 (Gu et al., 2014). Owing to the high prevalence in chicken flocks, the genotype S H9N2 viruses preferentially donate their internal genes to other AIVs in China (Gu et al., 2014). For instance, the six internal genes of the novel H7N9 influenza virus causing epidemic in human beings are derived from genotype S H9N2 virus in 2013 (Liu et al., 2013; Gu et al., 2014). Subsequently, a newly emerged avian H10N8 virus possesses internal gene cassette recruited from genotype S H9N2 AIV (Chen et al., 2014; Gu et al., 2014).

In fact, H9N2 and H5 AIVs co-exist in poultry in many regions in China, thus providing an ideal environment for the generation of novel AIVs by reassortment. Recently, various novel reassortant viruses whose HA gene originated from the A/goose/Guangdong/1/96-like (Gs/GD-like) lineage and the internal genes from H9N2 viruses were isolated in humans and birds (Zhao et al., 2012; Bi et al., 2016). We previously reported that the internal gene cassette of a natural reassortant H5N2 subtype AIV isolate (A/chicken/Hebei/1102/2010) with high pathogenicity for chickens and low pathogenicity for mice was from the genotype S H9N2 virus (Zhao et al., 2012). Moreover, H5N6 viruses internal gene cassette from the genotype S H9N2 virus was reported to be responsible for at least five among 17 H5N6 human cases (Bi et al., 2016). Hence, reassortment between H5 and H9 viruses can generate new viruses with unpredictable virulence and transmissibility, which may impose great threat to human health and poultry industry. However, the pathogenicity and transmission of the H5 reassortants with internal gene cassette of genotype S H9N2-origin in chickens and mice remains less clear.

In this study, four H5 reassortant viruses carrying the HA and NA genes from two subclade 2.3.2.1c and two subclade 2.3.4.4 of H5 viruses, respectively, and the remaining internal genes from an H9N2 virus of the predominant genotype S were generated by reverse genetics. Viral replication, polymerase activity and pathogenicity of these viruses in chickens and mice were evaluated. Additionally, transmission capacity of four H5 reassortant viruses in birds was also determined. Our study may help to further understand the specific effect of the internal gene cassette of genotype S H9N2 AIV on H5 viruses and provide valuable information for the control and prevention of the resultant recombinant viruses.

## Materials and Methods

### Ethics Statement

All experiments involving live viruses were carried out in a biosafety level 3 (BSL3) facility at Yangzhou University in accordance with the institutional biosafety manual.

Animals were housed in negative-pressure isolators with HEPA filters in a BSL3. The protocols for animal experiments were approved by the Jiangsu Administrative Committee for Laboratory Animals (approval number: SYXK-SU-2007-0005), and complied with the guidelines of Jiangsu laboratory animal welfare and ethics of Jiangsu Administrative Committee of Laboratory Animals.

### Viruses and Cells

Four H5 HPAIV strains including A/chicken/Anhui/QD1/2014 [QD/1 (H5N1)], A/goose/Jiangsu/QD5/2014 [QD/5 (H5N8)] (Li et al., 2016), A/chicken/Jiangsu/YB7/2015 [YB/7 (H5N1)] and A/Chicken/Jiangsu/k0402/2010 [CK/10 (H5N1)] (Hu et al., 2013), and one genotype S H9N2 virus A/Chicken/Jiangsu/CZ73/2014 [CZ/73 (H9N2)] were used in this study. QD/1 and QD/5 belong to subclade 2.3.4.4 while YB/7 and CK/10 belong to subclade 2.3.2.1c. The viruses propagated in 9- to 10-day-old specific-pathogen-free (SPF) chicken embryos.

Chicken embryo fibroblasts (CEF), and chicken fibroblast cell line (DF-1) cells were cultured in DMEM (Invitrogen) medium with 4% fetal bovine serum (FBS) (Gibco). Madin-darby canine kidney (MDCK), and human embryonic kidney (293T) cells were grown in DMEM containing 10% FBS. All cells were incubated at 37°C with 5% CO_2_. For virus inoculation experiments, DMEM medium supplemented with 1% FBS was used.

### Virus Rescue

Four reassortant viruses were generated by reverse genetics as described previously (Hao et al., 2016). The reassortants were generated through replacing the HA and NA genes of the H9N2 virus with several clades of H5 viruses. Viruses were propagated in 10-day-old SPF chicken embryos and were sequenced to ensure the absence of unwanted mutations. Sequence data were available in GenBank under the accession numbers (**Table 1**).

**Table 1:** The accession number of sequences of the parental viruses.

Virus	Accession number of sequence in GenBank
A/Chicken/Jiangsu/k0402/2010 (H5N1)	JQ638673- JQ638687
A/chicken/Jiangsu/YB7/2015 (H5N1)	MF673388-MF673393, KY776496- KY776497
A/chicken/Anhui/QD1/2014 (H5N1)	KY437804-KY437811
A/goose/Jiangsu/QD5/2014 (H5N8)	KT221063-KT221070
A/Chicken/Jiangsu/CZ73/2014 (H9N2)	KY776490- KY776495

### Viral Titration and Growth Curve

The titers of stock virus, expressed as 50% embryo infectious dose (EID_50_) and 50% tissue culture infectious dose (TCID_50_), were determined in chicken embryos and in MDCK and CEF cells as previously described (Hao et al., 2016). Briefly, 10-fold serial dilutions of the viruses were inoculated in chicken embryos or cells. After 4 days of incubation, TCID_50_ and EID_50_ titers were calculated using the Reed and Muench method (Reed and Muench, 1938).

Madin-darby canine kidney or CEF Cells were infected with the viruses at a multiplicity of infection (MOI) of 0.01 TCID_50_ Per cell. Supernatants were collected at 0.5, 1, 2, 3, and 4 day post-inoculation (dpi), and the virus titers were determined by TCID_50_ assay in MDCK cells as described above. Each experiment was performed in triplicate.

### Luciferase Assay

A minigenome assay was used to detect luciferase activities as reported previously (Bortz et al., 2011). Briefly, the p-Luci plasmid (p-Luci, carrying an IAV reporter minigenome in which the firefly luciferase gene is flanked by the non-coding regions of the NS gene from IAV, a truncated PolI promoter and the hepatitis delta virus ribozyme) (Ma et al., 2015) was transfected into 293T cells with Polyfect Transfection Reagent (Qiagen) together with the four pcDNA3.1(+) expression plasmids containing PB2, PB1, PA, and NP genes (400 ng each) from CK/10, YB/7, QD/5, QD/1, or CZ/73, and 40 ng internal control Renilla plasmid (an internal control plasmid to standardize transfection efficiency that encodes the Renilla luciferase protein). Likewise, as for DF1 cells, the four pcDNA3.1(+) expression plasmids of PB2, PB1, PA, and NP genes (400 ng each) were mixed with paviPolIT-Luc plasmid (paviPolIT-Luc, containing the firefly luciferase gene flanked by the non-coding regions of the NP gene of IAV is inserted into a pPolI plasmid housing the 250-nucleotide sequence of the avian polymerase I promoter) (Song et al., 2011) (400 ng) and the Renilla plasmid (80 ng) were co-transfected into DF1 cells. At 24 or 48 h post-transfection, cell lysates were prepared with Dual-Luciferase Reporter Assay System (Promega) and the firefly and Renilla luciferase activities were measured using GloMax 96 microplate luminometer (Promega). The ratio of the firefly luciferase activity value and Renilla luciferase activity value to represent RNP activity of the virus. All results are the means with SD from three independent experiments.

### Animal Experiments

#### Viral Pathogenicity and Transmission in Chickens

The highly pathogenic H5 AIV has the multiple basic amino acid at the HA cleavage site or possesses an intravenous pathogenicity index (IVPI) greater than 1.2, and one of the IVPI methods to determine pathogenicity in chickens is used according to the World Organisation for Animal Health (OIE) criteria (OIE, 2017). To determine viral pathogenicity in chickens, groups of ten 6-week-old SPF White Leghorn chickens were intravenously infected with 0.1 ml of a 1:10 dilution of the indicated viruses to measure IVPI, and the birds were observed for clinical signs or death for 10 days. To compare virus replication *in vivo*, groups of eleven 6-week-old SPF chickens were inoculated intranasally with 10^6.0^ EID_50_ of each virus. Three inoculated birds from each group were euthanized at 2 dpi, and gross lesions were recorded. At this time point, tissue samples (lung, brain, spleen, and kidney), trachea and cloacal swabs were collected for virus isolation. Another eight virus-infected chickens were monitored for mortality and morbidity for 14 days. Mean death time (MDT) was determined as the mean time (d) for the virus to kill chickens within the observation period. For histopathological evaluation, the lung specimen from each group was fixed in 10% buffered formalin, embedded in paraffin, and sectioned at 5 μm. Three tissue sections from each lung, and five sections per bird were stained with hematoxylin-eosin (H&E). This experiment was performed by three trained pathologists blinded to the treatment groups. The tissue pathological changes were observed and scored with an Olympus microscope (Olympus Optical Co., Ltd.). Criteria for grading lung pathological changes as described previously (Han et al., 2014): Grade 0 = no obvious pathological changes; Grades 1–3 = light inflammatory cells infiltration, light hemorrhage, cell shedding; Grades 4–5 = inflammatory cells infiltration, hemorrhage, vasculitis or bronchiolitis, cell apoptosis and necrosis; Grades 6–10 = severe inflammatory cells infiltration, severe hemorrhage, vasculitis or bronchiolitis, obvious edema, cell apoptosis and necrosis. In addition, the levels of cytokine and chemokine in the lung were determined by ELISA (Bio-Swamp, Wuhan, China).

Transmission experiments were designed as previously described (Zhang et al., 2009). Briefly, for each virus, nine of 6-week-old SPF chickens were divided into three groups. The three chickens were inoculated intranasally with 10^4.0^ EID_50_ of each virus, as the direct inoculation group. The three chickens were placed in the same cage with the inoculated chickens at 30 min after inoculation of the infected group, as the physical contact group. The three chickens was placed in a cage directly adjacent to the direct inoculation group with a distance of 50 cm between cages, as the aerosol contact group (Zhong et al., 2014). Oropharyngeal swabs were collected from all chickens at 3, 5, 7, and 9 dpi for virus titration in chicken embryos. Meanwhile, all birds were observed daily for clinical symptoms for 14 days.

#### Viral Pathogenicity in Mice

To determine the 50% mouse lethal dose (MLD_50_) of the viruses, groups of 5 (6-week-old) female BALB/c mice were inoculated intranasally with serial dilutions of virus and observed the morbidity and mortality for 14 days as previously described (Hao et al., 2016). To analyze viral replication *in vivo*, groups of mice were infected of 10^6.0^ EID_50_ of the indicated viruses. At 3 and 5 dpi, three mice from each group were euthanized and the kidney, spleen, brain, and lung were harvested for virus titration in eggs. Samples were homogenized in 1 ml of cold PBS and were infected in chicken embryos and titers were calculated using the Reed and Muench method (Reed and Muench, 1938). The threshold of detection was 10^1.0^ EID_50_ per ml for each tissue. Additionally, lungs were treated as described above for histopathological examination. The levels of cytokine and chemokine in the lung of mouse were measured using ELISA (Bio-Swamp, Wuhan, China).

### Statistical Analysis

The Independent-Samples *t*-Test was used for data analysis. A value of *P* < 0.05 was considered as statistically significant. Statistical analyses were performed using the SPSS Statistics software (IBM company, SPSS 19.0).

## Results

### Generation of H5 Influenza Viruses with Internal Gene Cassette from the Genotype S H9N2 Virus

Four H5 reassortants harboring the surface genes from CK/10 (H5N1), YB/7 (H5N1), QD/1 (H5N1), QD/5 (H5N8) viruses and the internal genes from CZ/73 (H9N2) were successfully generated by reverse genetics (**Table 2**). The CK/S, Y7/S were successfully rescued in one time, whereas Q1/S and Q5/S were generated in two and three experiments, respectively (**Table 2**). To investigate the properties of the rescued reassortants and the H5 parental viruses, EID_50_ and TCID_50_ were assessed. As shown in **Table 2**, the reassortants (CK/S, Y7/S, Q1/S, and Q5/S) and the parental viruses (CK/10, YB/7, QD/1, QD/5, and CZ/73) infected efficiently both in MDCK, CEF cells and chicken embryos. Viruses grew to similar titers in MDCK and CEF cells and chicken embryos, from 10^6.0^ to 10^7.8^, 10^6.4^ to 10^8.6^, 10^7.2^ to 10^8.8^, respectively. However, Q5/S showed lower infectivity in CEF cell with a TCID_50_ of 10^4.5^. Overall, these results indicate that the internal gene cassette from the genotype S H9N2 have high genetic compatibility with the surface genes of the tested H5 viruses.

**Table 2:** Infectivity of the parental and reassortant viruses.

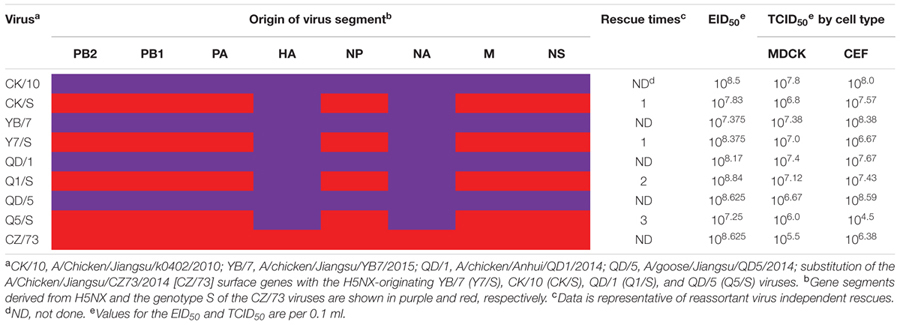

### Internal Gene Cassette of the Genotype S H9N2 Virus Decreases Replication of H5 Recombinants *in Vitro*

**FIGURE 1:**
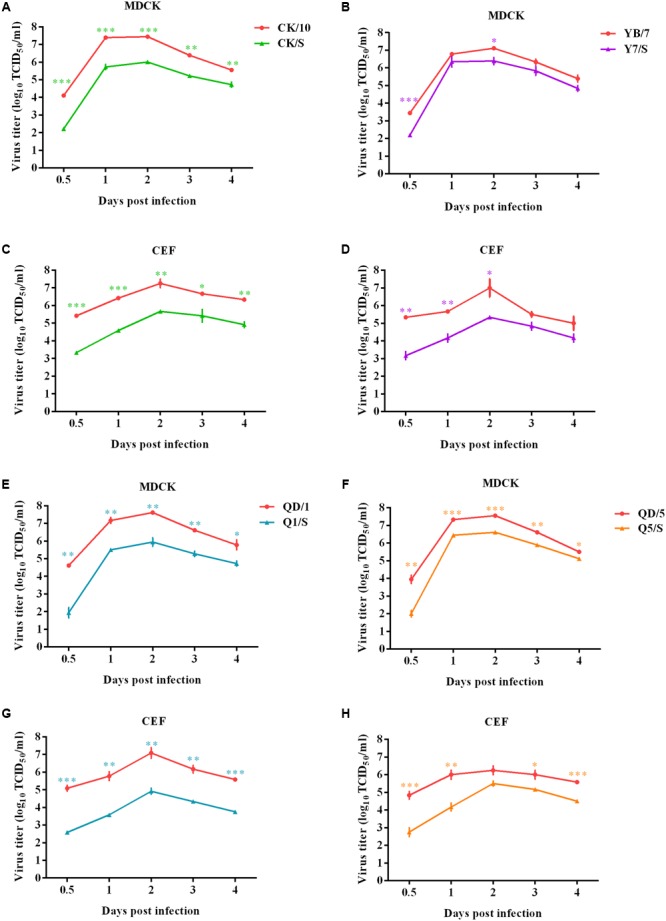
Multiple-cycle growth curves of the H5 viruses. The replication abilities of the viruses were measured by multiple-cycle growth curve analysis on MDCK and CEF cells. Virus titers were determined in MDCK at indicated time points. Data represent the mean ± SD of three independent infections. ^∗^, compared to the corresponding parental H5 virus. (^∗^*P* < 0.05; ^∗∗^*P*<0.01; ^∗∗∗^*P* < 0.001). Panels **(A)** and **(C), (B)** and **(D), (E)** and **(G), (F)** and **(H)** show the growth curves of CK/10 and CK/S, YB/7 and Y7/S, QD/1 and Q1/S, QD/5 and Q5/S infected MDCK and CEF cells, respectively.

To evaluate whether the internal gene cassette of the genotype S H9N2 virus affected replication of the H5 viruses in avian and mammalian cells, we compared kinetics of replication of the viruses in both CEF and MDCK cells. As a result, the recombinants presented significantly lower replication activity in both MDCK (**Figures 1A,B,E,F**) and CEF (**Figures 1C,D,G,H**) cells at several time points as compared to their respective parental viruses (**Figure 1**). Of noted, the recombinants CK/S and Q1/S presented significantly lower viral titers at all the tested time points both in MDCK and CEF cells when compared with the corresponding parental viruses. These data show that the internal gene cassette of the genotype S H9N2 virus decreases the replication ability of the H5 viruses in mammalian and avian cells.

### Internal Gene Cassette of the Genotype S H9N2 Virus Decreases Polymerase Activity of H5 Recombinants

**FIGURE 2:**
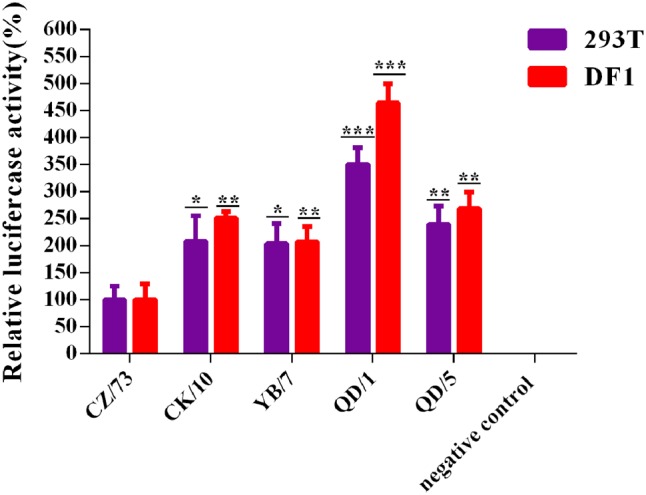
Polymerase activities of reconstituted RNP complex by minigenome assay. Human 293T or avian DF-1 cells were co-transfected with plasmids expressing PB2, PB1, PA, and NP from CK/10, YB/7, QD/5, QD/1, or CZ/73 virus, together with a firefly luciferase reporter plasmid, and a Renilla luciferase reporter plasmid (internal control). After 24 h or 48 h, cell lysates were used to measure firefly and Renilla luciferase activities. Values shown represent the means ± SD deviations of the results of three independent experiments and are standardized to those of CZ/73 (100%). The value of each recombinant virus was compared with that of the corresponding parental virus (^∗^*P* < 0.05, ^∗∗^*P* < 0.01, ^∗∗∗^*P* < 0.001).

To investigate the relationship between RNP activities and the replication ability of these reassortants, we determined the activities of the combined RNP complexes from the CK/10 (H5N1), YB/7 (H5N1), QD/1 (H5N1), QD/5 (H5N8), or CZ/73 (H9N2) virus gene segments by measuring the luciferase activity in 293T and DF-1 cells. As shown in **Figure 2**, the RNP activity of QD/1 (H5N1) were 364 and 250% higher than those of CZ/73 (H9N2) in DF-1 and 293T cells. Activity of QD/5 (H5N8), CK/10 (H5N1) and YB/7 (H5N1) RNP complexes were 168% (*P* < 0.01), 151% (*P* < 0.01), and 107% (*P* < 0.05) higher than that of CZ/73 (H9N2) in DF-1 cells, respectively. Additionally, QD/5 (H5N8), CK/10 (H5N1), and YB/7 (H5N1) RNP activities were 138% (*P* < 0.01), 108% (*P* < 0.05), and 103% (*P* < 0.05) higher than that of CZ/73 (H9N2) in 293T cells, respectively. Collectively, CK/10 (H5N1), YB/7 (H5N1), QD/1 (H5N1), and QD/5 (H5N8) complexes showed higher RNP activities than that of CZ/73 (H9N2). Our findings suggest that the internal gene cassette of the genotypes S H9N2 virus significantly decreases the RNP activity of the H5 recombinants.

### Internal Gene Cassette of the Genotype S H9N2 Virus Decreases Pathogenicity and Replication of H5 Viruses in Chickens

To examine the pathogenicity of these H5 viruses in chickens, we determined the replication and IVPI of the viruses in chickens. The IVPIs of all the viruses were over 2.4, indicating that they were highly pathogenic in chickens. As shown in **Table 3**, the IVPI of CK/S, Y7/S, and Q5/S were 0.1 lower than that of their parental viruses, and Q1/S showed a decrease of 0.5 in relative to QD/1. The IVPI result suggested that the H5 recombinants had lower virulence in chickens when compared to their parental viruses. Intriguingly, MDTs of the group infected with parental viruses were about 2 days, while the chickens infected with four H5 reassortants were over 5 days. Especially, the Q1/S virus resulted in a mortality of 50% and an MDT of 8.75 days, whereas the QD/1 virus brought about a higher mortality (100%) and a shorter MDT (2.13 days) (**Table 3**). Therefore, MDTs and survival rates were significantly different (*P* < 0.001) between the H5 reassortants and the parental viruses.

**Table 3:** Pathogenicity of the parental and reassortant viruses in SPF chickens.

Virus name	Number of sick/dead/total^a^	Mean days of death MDT (days)^b^	IVPI^c^
CK/10	8/8/8	2.0	3.0
CK/S	8/8/8	5.63**	2.9
YB/7	8/8/8	2.0	3.0
Y7/S	8/8/8	5.63**	2.9
QD/1	8/8/8	2.13	2.9
Q1/S	5/4/8	8.75**	2.4
QD/5	8/8/8	2.25	2.8
Q5/S	8/7/8	6.0**	2.7

*^a^Morbidity, mortality, and total resulting from intranasally inoculation of 6-week-old SPF chickens were infected intranasally with 10^*6.0*^ EID_*50*_ of each virus. ^b^Mean days of death, MDT resulting from intranasally inoculation of 6-week-old SPF chickens were infected intranasally with 10^*6.0*^ EID_*50*_ of each virus. ^c^Intravenous pathogenicity index, IVPI. ^∗∗^*P* < 0.01 compared with the value for the corresponding parental H5 virus.*

**FIGURE 3:**
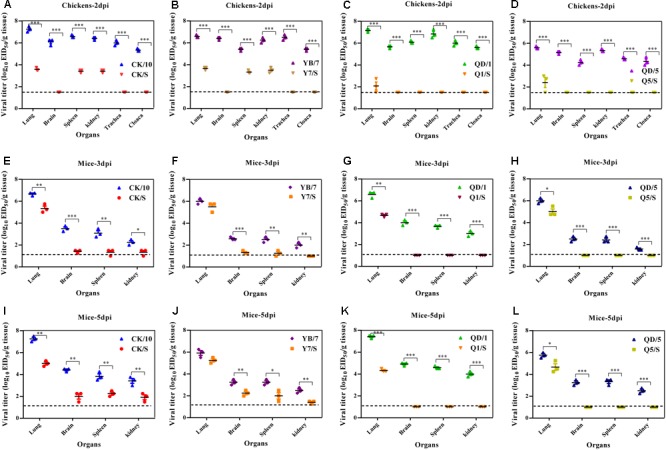
Replication of the H5 viruses *in vivo*. **(A–D)** Viral replication in chickens. Groups of three 6-week-old SPF chickens were intranasally infected with 10^6.0^ EID_50_ of the indicated viruses. At 2 dpi, three birds of each group were euthanized to collect organs for virus titration in eggs. **(E–L)** Viral replication in mice. Groups of nine 6-week-old female BALB/c mice were intranasally infected with 10^6.0^ EID_50_ of the indicated viruses. At 3 and 5 dpi, three mice of each group were euthanized to collect organs for virus titration in eggs. Viral loads are expressed as the mean ± SD. ^∗^*P* < 0.05, ^∗∗^*P* < 0.01, ^∗∗∗^*P* < 0.001 compared to the corresponding parental virus. The detection limit for virus titers in chickens is 10^1.5^ EID_50_ per g for each tissue and in mice is 10^1.0^ EID_50_ per ml.

To examine the replication of the viruses in chickens, groups of three 6-week-old SPF chickens were intranasally inoculated with 10^6^ EID_50_ of the viruses. Three chickens from each group were killed at 2 dpi, and their organs were collected for virus titration. At this time point, the parental viruses could be detected in the lungs, brains, spleens, kidneys from three birds, whereas Y7/S, CK/S could be detected in the lungs, spleens, kidneys from three birds (**Figures 3A,B**) and Q1/S, Q5/S could only be detected in the lungs from two out of three birds (**Figures 3C,D**). In addition, parental viruses were recovered successfully in the trachea and cloacal swabs of the infected birds. Viral titers were higher in the trachea than in the cloacal swabs at this time point. However, we failed to detected virus from trachea and cloacal samples of the H5 reassortants-infected birds at this time point (**Figures 3A–D**). Moreover, a significantly higher virus load was observed in the lung, brain, spleen, and kidney of the parental viruses-infected chickens compared to those of the H5 reassortants (**Figures 3A–D**).

**FIGURE 4:**
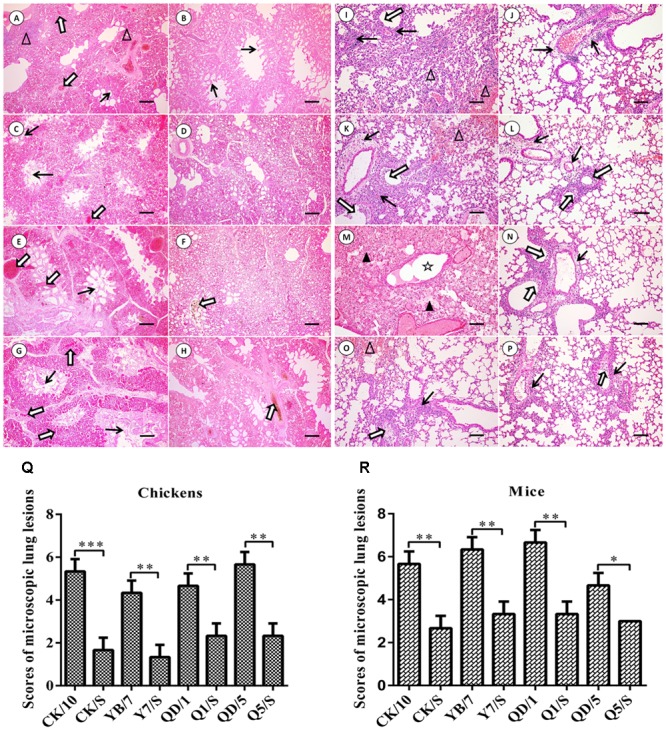
Histopathological changes in H&E-stained lung tissues of the H5 virus-infected SPF chickens **(A–H)** and BALB/c mice **(I–P)**. **(A–H)** Show the H&E-stained lung sections (magnification, 10×) at 2 dpi of the CK/10, CK/S, YB/7, Y7/S, QD/1, Q1/S, QD/5, Q5/S infected birds, respectively. Bronchial epithelial cell desquamation, lymphocytes infiltrates around lung lobular, and interstitial congestion, erythrocytes within alveolar spaces are denoted with thick black arrows, white triangles, and thick white arrows, respectively (scale bar: 200 μm). (I–P) Show the H&E-stained lung sections (magnification, 20×) at 5 dpi of the CK/10, CK/S, YB/7, Y7/S, QD/1, Q1/S, QD/5, Q5/S infection groups, respectively. Inflammatory cell infiltration, deciduous epithelium mucosae and hemorrhage, and inflammatory cells infiltrates in alveolar, serous and inflammatory cells within alveolar spaces and bronchial lumen expansion are denoted with thick black arrows, thick white arrows, white triangles, black triangles and five-pointed star, respectively (scale bar, 100 μm). **(Q,R)** Show the scores of pathological changes in recombinants and parental viruses infection groups birds or mice. ^∗^*P* < 0.05, ^∗∗^*P* < 0.01, and ^∗∗∗^*P* < 0.001 between recombinants and parental viruses infection groups.

We further systematically compared the virus-induced lung histological changes between the parental viruses and the recombinants. As shown in **Figures 4A–H**, the lungs of the H5 parental viruses-infected chickens showed severe bronchopneumonia, characterized by severe bronchopneumonia and interstitial pneumonia, bronchial epithelial cell desquamation, lymphocytes infiltrates around lung lobular, interstitial congestion, erythrocytes within alveolar spaces. By contrast, mild bronchopneumonia and mild desquamation of bronchial epithelial cells were seen in the lung of the H5 recombinants infected-birds. Scores of microscopic lung lesions are shown in **Figure 4Q**. Taken together, these results suggest that the internal gene cassette of the genotype S H9N2 virus decreases pathogenicity of the H5 viruses in chickens.

### Transmission of H5 Reassortant Viruses in Chickens

Because the MDTs of the H5 reassortants were longer than the parental viruses, we then investigated the transmission of four H5 reassortants in chickens. All reassortants viruses were detected in oropharyngeal swabs from the directly inoculated birds (**Table 4**). As a result, during the observation period, all chickens from the physical contact group that were placed in the same cage with the CK/S or Y7/S-infected chickens, died within 9 dpi. Viruses were detected in oropharyngeal swabs of the CK/S or Y7/S of the physical contact birds at 3, 5, and 7 dpi and the mean titers were from 10^3.25^ to 10^4.0^ EID_50_, 10^3.33^ to 10^3.92^ EID_50_, respectively (**Table 4**). The physical contact birds housed with the Q1/S survived on 9 dpi, shed with titers detected at a range from 10^3.25^ to 10^3.67^ EID_50_ and showed seroconversion at 14 dpi. One of three physical contact birds housed with the Q5/S died on 9 dpi and the rest of the physical contact birds seroconverted on 14 dpi (**Table 4**). The virus shedding was tested from oropharyngeal swabs of the Q5/S of the physical contact birds with titers detected at a range from 10^3.08^ to 10^3.88^ EID_50_ on 9 dpi (**Table 4**). However, no virus from airborne transmission chickens of four reassortants could be detected in oropharyngeal during the observation period except the CZ/73 (H9N2) group (**Table 4**). All together, these results indicate that four reassortants are transmitted to direct contact group but fail to be transmitted efficiently by aerosol way.

**Table 4:** Transmission of the H5 reassortant viruses in chickens.

Strains	Method of transmission	Days post-inoculation (log_10_EID_50_/0.1 ml) ± SD^a^			
		oropharyngeal swabs	Oropharyngeal swabs	Oropharyngeal swabs	Oropharyngeal swabs
		
		3	5	7	9
	Direct inoculation	3.75 ± 0.25 (2/3)	4.67 ± 0.28 (2/2)	3.88 (1/1)	ND^b^
CK/S	Physical contact	3.25 ± 0.27 (1/3)	4.0 ± 0.13 (2/3)	3.75 ± 0.18 (1/2)	ND
	Aerosol contact	1.5 ± 0 (0/3)	1.5 ± 0 (0/3)	1.5 ± 0 (0/3)	1.5 ± 0 (0/3)
	Direct inoculation	3.58 ± 0.17 (3/3)	4.25 ± 0.17 (3/3)	3.88 (1/1)	ND
Y7/S	Physical contact	3.33 ± 0.27 (1/3)	3.92 ± 0.13 (2/3)	3.58 ± 0.18 (1/2)	ND
	Aerosol contact	1.5 ± 0 (0/3)	1.5 ± 0 (0/3)	1.5 ± 0 (0/3)	1.5 ± 0 (0/3)
	Direct inoculation	3.25 ± 0.13 (2/3)	4.25 ± 0.28 (3/3)	3.25 ± 0.11 (2/3)	3.25 ± 0.11 (2/3)
Q1/S	Physical contact	3.25 ± 0.25 (1/3)	3.58 ± 0.13 (2/3)	3.67 ± 0.13 (1/3)	3.58 ± 0.17 (1/3)
	Aerosol contact	1.5 ± 0 (0/3)	1.5 ± 0 (0/3)	1.5 ± 0 (0/3)	1.5 ± 0 (0/3)
	Direct inoculation	3.33 ± 0.17 (2/3)	4.5 ± 0.17 (3/3)	3.42 ± 0.13 (2/3)	3.25 ± 0.25 (2/2)
Q5/S	Physical contact	3.25 ± 0.25 (1/3)	3.88 ± 0.13 (2/3)	3.33 ± 0.17 (1/3)	3.08 ± 0.11 (1/3)
	Aerosol contact	1.5 ± 0 (0/3)	1.5 ± 0 (0/3)	1.5 ± 0 (0/3)	1.5 ± 0 (0/3)
	Direct inoculation	3.38 ± 0.23(3/3)	4.0 ± 0.23 (3/3)	3.08 ± 0.17 (2/3)	1.5 ± 0 (0/3)
CZ/73	Physical contact	3.75 ± 0.25 (3/3)	4.38 ± 0.13 (3/3)	3.75 ± 0.25 (3/3)	1.5 ± 0 (0/3)
	Aerosol contact	3.83 ± 0.23 (1/3)	4.5 ± 0.25 (2/3)	3.5 ± 0.15 (3/3)	2.38 ± 0.17 (2/3)

*^a^For statistical purposes, a value of 1.5 was assigned if virus was not detected from the undiluted sample in three chicken embryos. ^b^ND, not detected. Chickens all died.*

### Internal Gene Cassette of the Genotype S H9N2 Virus Decreases Pathogenicity and Replication of H5 Viruses in Mice

**FIGURE 5:**
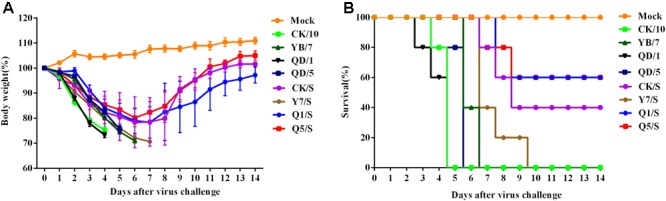
Pathogenicity of the H5 viruses in mice. Five 6-week-old female BALB/c mice per group were intranasally inoculated with 10^6.0^ EID_50_ (in 50 μl) of the indicated viruses or mock inoculated (Mock). Body weight **(A)** and survival rate **(B)** was monitored daily for 14 days. Mice that lost more than 25% of their initial weight were euthanized. The percentage weight from each group and each time point are presented as means ± SD.

To investigate the pathogenicity of these H5 viruses in mouse models, we then determined the MLD_50_ of the recombinants in BALB/c mice. As shown in **Table 5**, we found that all of the H5 reassortants showed decreased virulence in mice than their respective parental viruses. The parental CK/10, QD/1, and QD/5 viruses were highly pathogenic in mice (MLD_50_: 10^0.65^, 10^0.5^, and 10^2.5^ EID_50_). In contrast, the H5 reassortants CK/S, Q1/S, and Q5/S viruses were moderately pathogenic for mice, with MLD_50_ values of 10^5.5^, 10^5.8^, and 10^6.0^ EID_50_, respectively. Moreover, Q1/S showed the greatest attenuation, with a 10^5.3^-fold decrease in MLD_50_. In addition, mice infected with these parental viruses resulted in >25% body weight loss and 100% mortality in mice in a dose of 10^6.0^ EID_50_ early before 6 dpi (**Table 5**). By contrast, mice infected with Q1/S, Q5/S, and CK/S viruses lead to two, two, three of five observed mice died, but the surviving mice recovered from inoculation by 8 dpi and experienced 14.8–17.4% weight loss, and the mean survival time (MST) 9.4–11.2 days (**Table 5**). The mortality rates of 40, 40, and 60 was observed in mice infected with Q1/S, Q5/S, and CK/S (**Figure 5**). Furthermore, Y7/S resulted in 19.6% weight loss, 100% mortality and the 6.2 days of MST (**Table 5**). Taken together, the internal gene cassette of the genotype S H9N2 virus significantly decreases the virulence of the H5 recombinants in mice.

**Table 5:** Pathogenicity of the parental and reassortant viruses in BALB/c mice.

Virus name	MLD_50_ (EID_50_)^a^	Weight loss (%)^b^	MST (days)^c^
CK/10	10^0.65^	>25	3.8
CK/S	10^5.5^	17.4^∗∗^	9.4*
YB/7	10^3.83^	>25	5.4
Y7/S	10^5.27^	19.6^∗^	6.2*
QD/1	10^0.5^	>25	3.4
Q1/S	10^5.8^	13.6^∗∗^	11.2**
QD/5	10^2.5^	>25	4.6
Q5/S	10^6.0^	14.8^∗∗^	11.2**

*^a^Values are EID_*50*_ equivalents. ^b^Weight loss, maximum mean weight loss was determined from five mice (percentage weight loss relative to 0 dpi) following infection with 10^*6*^ EID_*50*_ of virus. ^c^MST, mean survival time of mice infected with 10^*6*^ EID_*50*_. ^∗^*P* < 0.05, ^∗∗^*P* < 0.01 compared with the value of the corresponding parental H5 virus.*

We next evaluated the replication ability of the viruses *in vivo*. All the parental viruses, and the reassortants (CK/S, Y7/S) could be detected in the lungs, brains, spleens, and kidneys, whereas Q1/S, Q5/S could only be detected in the lungs (**Figures 3E–L**). Additionally, significantly higher virus yields in the lungs of CK/10, QD/1, and QD/5 viruses-infected mice were observed compared to those of CK/S, Q1/S, and Q5/S viruses on 3 and 5 dpi (**Figures 3E–L**). In addition, virus titers in the brains, spleens, and kidneys of CK/10, YB/7 viruses-infected birds were dramatically higher than those of CK/S, Y7/S viruses on 3 and 5 dpi (*P* < 0.01) (**Figures 3E,F,I,J**). Notably, Q1/S and Q5/S viruses showed no replication in the brains, spleens, and kidneys. Collectively, these results suggest that the internal gene cassette of the genotypes S H9N2 virus significantly decreases the replication of the H5 recombinants in mice.

Hematoxylin-eosin staining of lung tissues was performed for histopathological evaluation. As shown in **Figures 4I–P**, the parental H5 viruses of this group resulted in severe interstitial pneumonia and bronchopneumonia at 5 dpi, characterized by edema, hemorrhages, desquamation, necrosis, and extensive infiltration of inflammatory cells. By contrast, mild to moderate bronchopneumonia was observed in the H&E-stained lung tissues of mice infected with the reassortant H5 viruses of this group. Scores of lung histopathological lesions are shown in **Figure 4R**. Collectively, these results indicate that the internal gene cassette of the genotypes S H9N2 virus dramatically decreases the pathogenicity of the H5 recombinants in mice.

### Internal Gene Cassette of the Genotype S H9N2 Virus Decreases the Cytokine Responses in the Lung

**FIGURE 6:**
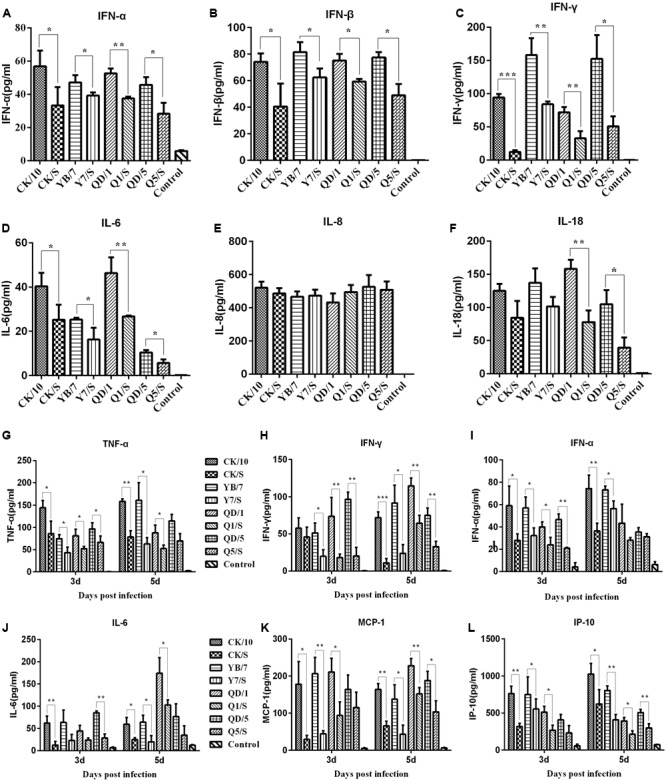
Cytokines and chemokines expression in SPF chicken lung **(A–F)** and the mouse lung **(G–L)**. Groups of three 6-week-old SPF chicken or Groups of 6 6-week-old female BABL/c mice were intranasally infected with 10^6.0^ EID_50_ of the indicated virus or received PBS intranasally (Control). On 2 dpi, three chicken of each group were euthanized, the lungs were collected for homogenate preparation. As for mice, on 3, and 5 dpi, three mice of each group were euthanized, the lungs were also collected for homogenate preparation. Lung concentrations of cytokines were measured by ELISA. Cytokine or chemokine expression was expressed as the mean concentration ± SD. ^∗^*P* < 0.05, ^∗∗^*P* < 0.01, ^∗∗∗^*P* < 0.001 indicates significant difference between the recombinant virus and the parental virus.

To better understand the role of cytokine expression in the induction of the different lung pathological manifestations caused by the recombinants and the parental viruses, we then systematically investigated the production of some representative cytokines and chemokines in the lungs of the virus-infected chickens and mice. In chickens, we analyzed six antiviral proinflammatory cytokines from the lungs of the virus-infected chickens at 2 dpi. As shown in **Figures 6A–F**, the parental viruses elicited higher levels of interleukin 6 (IL-6), interferon α (IFN-α), interferon β (IFN-β), and interferon γ (IFN-γ) compared to the recombinant-infected lungs. However, the recombinants and the parental viruses elicited similar cytokine responses in terms of interleukin 8 (IL-8) expression (**Figures 6E,F**). In addition, significantly higher levels of interleukin 18 (IL-18) genes were induced by QD/1 than Q1/S, QD/5 than Q5/S, whereas there were similar levels in this gene induction between CK/10 and CK/S, YB/7 and Y7/S.

In mice, 6 antiviral proinflammatory cytokines were analyzed in the virus-infected mouse lungs. Significantly higher levels of cytokines tumor necrosis factor α (TNF-α), IL-6, IFN-γ, IFN-α, monocyte chemotactic protein 1 (MCP-1), interferon-inducible protein 10 (IP-10) were detected in the lungs of the parental viruses-infected mice than in those inoculated with the recombinants at 3 and 5 dpi (**Figures 6G–L**). Thus, these results indicate that internal gene cassette of the genotype S H9N2 virus dramatically decreases cytokine and chemokine response in both chickens and mice lungs.

## Discussion

Asian lineage of H5 influenza virus continues to evolve and may generate a phenotype with pandemic potential. Especially, subtype H5 influenza virus is prone to reassorting with poultry-derived H9N2 viruses, such as novel reassortant HPAI H5N2, and H5N6 viruses, with considerable threat to agriculture and human health (Xu et al., 2015; Bi et al., 2016). Moreover, many of the currently circulating novel reassortant contain the genotype S H9N2 internal gene cassette, such as the 2013 novel H7N9, H7N7, and H10N8 viruses (Gu et al., 2014; Liu et al., 2014). Internal gene cassette of the genotype S H9N2 consists of the PB2 and M from A/quail/Hong Kong/G1/97-like, the PA, PB1, and NP from A/chicken/Shanghai/F/98-like viruses, and NS from the A/chicken/Beijing/1/94(BJ/94-like) (Gu et al., 2014). Based on these findings, it is highly probable that the genotype S H9N2 internal gene cassette is a stable combination that preferentially reassort with different surface genes (Liu et al., 2014). Additionally, accumulating evidence suggests that combinations of AIVs internal genes are considered to contribute to viral virulence (Sun et al., 2011; Bi et al., 2015; Su W. et al., 2015; Hao et al., 2016). However, the specific effect of the genotype S H9N2 virus internal gene cassette on the pathogenicity of H5 HPAIV in chickens and mice remains unclear.

In order to investigate whether the genotype S H9N2 virus internal gene cassette affected the pathogenicity of H5 HPAIV, one H9N2 and four H5 viruses were selected as parental viruses. There are three major considerations for choosing these viruses in our study: (i) Epidemic strains, phylogenetic analysis of the HA genes revealed that CK/10 (H5N1) and YB/7 (H5N1) belong to subclade 2.3.2.1c while QD/1 (H5N1) and QD/5 (H5N8) belong to subclade 2.3.4.4, both of which are currently the predominant subclades of H5 HPAIVs circulating in China. The CZ/73 (H9N2) virus belongs to genotypes S, which is the predominant genotype in the field throughout China. (ii) In previous studies, we have collected a very large number of biological materials of these viruses (Hu et al., 2012; Li et al., 2016). (iii) Reassortment, they have undergone genetic reassortment to produce naturally occurring recombinant viruses between H9 and H5 viruses (Su S. et al., 2015). Four H5 reassortants carrying the surface genes from the H5 viruses and the remaining internal genes from the genotype S H9N2 virus were generated. The *in vitro* and *in vivo* findings clearly demonstrated that all the H5 reassortant viruses showed attenuated pathogenicity in both chickens and mice. Of note, the MDTs of the reassortants in chickens and mice were all much longer than the parental viruses. Moreover, these hybrid viruses could transmit to birds by direct contact, but they failed to transmit among birds by airborne route.

Comparing with the parental H5 viruses, all of four generated H5 reassortants with the internal gene cassette from genotype S H9N2 viruses showed a reduction of virus growth in MDCK and CEF cells, respectively (**Figure 1**). Furthermore, a remarkably lower luciferase activity of the H9N2-derived RNP complexes was also detected in both 293T and DF-1 cells in contrast to either of the H5NX-derived ones (**Figure 2**). Since RNP compatibility and activity are closely related to viral reassortment, replication, and pathogenicity (Sun et al., 2011; Bi et al., 2015; Hao et al., 2016), we speculate that the relatively poor polymerase activity of CZ/73 (H9N2) was associated with the lower replication ability of the H5 reassortants in CEF and MDCK cells. In addition, such H9N2 virus internal genes mediated low polymerase activity may yet contribute to the decreased virulence of the H5 reassortants in chickens and mice.

Despite highly pathogenic to chickens as evidenced by the IVPI value, each of four H5 reassortants showed obviously longer MDTs than the corresponding parental H5 viruses (**Table 3**), which may facilitate the longer virus shedding and circulation to pose a persistent threat to poultry. Different from H5 HPAI field isolates that generally fail to transmit between chickens in aerosol just with few variants showing contact transmission (Jiao et al., 2016; Cui et al., 2017), H9N2 subtype AIVs possess both horizontal contact and aerosol transmissibility in poultry (Zhang et al., 2009; Zhong et al., 2014). In addition, we have previously demonstrated that both surface and internal genes are critical for airborne transmission for H9N2 virus in chickens (Zhong et al., 2014). In the present study, although virus shedding was observed in all of the physically contact chickens in the four groups inoculated with H5 reassortants containing whole internal genes from H9N2 (**Table 4**), transmission between birds by aerosol route was not detected except the CZ73 (H9N2) group (**Table 4**), indicating that the envelop gene segments of H9N2 may contribute more critically to the acquisition of aerosol transmission. However, since all of the parental H5 HPAI viruses were lethal to chickens within 2 days even at the low inoculation dosage of 10^3.0^ EID_50_ (data not shown), we failed to include those viruses in the transmission experiment. Therefore, whether the internal gene cassette of H9N2 subtype AIVs enhance the contact transmissibility of reassortant H5 viruses as inferred by our results yet needs further investigation.

As previously documented, internal genes from LPAIV donor like H9N2 could attenuate the virulence of generated reassortants (Sun et al., 2011; Su W. et al., 2015). For example, the PB2 gene from H9N2 was involved in attenuating the pathogenicity of H7N9 virus in mice (Su W. et al., 2015). Sun et al. (2011) also found that the PB2, PB1, NP, M, and NS genes of H9N2 decreased the virulence of 2009 pandemic H1N1 virus in mice. In this study, consistent with the results in chickens, the H5 reassortant viruses also displayed significantly attenuation in pathogenicity in mice compared with the parental viruses (**Table 5**). Of note, the attenuated H5 reassortants could easily infected mice, implying that these viruses were prone to circulating in a mammalian host and may evolve via adaptive mutation or further reassortment capable of posing a potential threat to human health. For example, our group previously isolated a natural H5N2 virus, A/chicken/Hebei/1102/2010 (HB10), which exhibited high virulence in chickens but was low pathogenic to mice (Zhao et al., 2012). Intriguingly, HB10 possessed the HA gene belongs to H5 viruses and the remaining genes from the genotype S H9N2 virus. Therefore, our results are in line with the findings of the pathogenicity of the natural recombinant H5N2 (HB10) in chickens and mice. Further studies by Li et al. (2014) showed that the HB10 virus was mutated and became more virulent to mice after fifteen serial lung-to-lung passages in mice. Thus, we surmised that the H5N2 recombinants contained genotype S H9N2 subtypes AIV internal gene cassette may pose a potential threat for human health through acquiring adaptive mutation or further reassortment (Li et al., 2015).

Among the 254 (*n* = 2^8^-2) theoretically generated genome constellations of the two parental H5 and H9 AIVs in the present study, only the combination pattern that containing all the six internal genes from the H9 virus while the two surface genes from H5 virus was investigated systematically to mainly focus on the biological effect especially pathogenicity variation of the internal gene cassette of genotype S H9N2 to reassortant H5 AIVs. Previous studies have shown that the highlighted contribution of a single gene segment to pathogenicity could be influenced by the other seven segments of AIV. For example, Sun et al. (2011) found that the H9 reassortants possessing single PA gene from the pandemic H1N1/2009 virus could augment the virulence in mice whereas the ones possessing all the six internal genes from the pandemic H1N1/2009 virus displayed a lower pathogenicity than the parental H9 viruses. Additionally, in the present study, we found that the H5 reassortants containing the whole internal gene cassette from genotype S H9N2 virus exhibited evidently lower virulence in both chickens and mice than their parental H5 viruses (**Tables 3, 5**), although we had previously demonstrated that the single PA or NP gene of genotype S H9N2 virus dramatically increased the virulence of the H5N1 recombinants in mice (Hao et al., 2016). In fact, multiple virulence factors were relevant to the pathogenesis of the AIV (Chen et al., 2007). Su W. et al. (2015) evaluated the pathogenicity of 63 H7N9 reassortants containing HA and NA from a chicken-origin H7N9 epidemic virus plus six other genes from H7N9 or H9N2 virus and found that 13 reassortants showed pathogenicity lower than that of the parent in mice. Chen et al. (2008) reported that the reassortants between avian H5N1 and human H3N2 viruses and evaluated the pathogenicity of 63 reassortants with the surface genes from an avian H5N1 virus and the internal genes from H5N1 or H3N2 parental virus. Their study revealed that all these reassortants have diminished virulence compared to the parental avian H5N1 virus (Chen et al., 2008).

In summary, our results demonstrated the high compatibility between the internal genes of the genotype S H9N2 viruses and the surface genes of the H5 viruses and that H5 reassortant viruses showed attenuated pathogenicity and replication in mice and chickens. In addition, the MDTs of the H5 recombinants were longer than those of their respective parental viruses. The results suggest that the H5 recombinants contained genotype S H9N2 subtypes AIV internal gene cassette might circulate in avian or mammalian host, evolve via reassortment or mutation, which is a potential threat to poultry industry and public health. Hence, our work highlights the necessity for strengthening surveillance to counteract the potential pandemic threat posed by the H9 and H5 reassortant viruses.

## Author Contributions

XH, JH, MG, and XfL conceived and designed this research. XH, JW, and XlL performed experiments. XH and XfL wrote the manuscript. XW, MG, ZH, ZG, DL, JL, XwL, SH, XX, DP, and XJ contributed reagents/materials/analysis tools. XH, JH, MG, and XfL revised the manuscript. All authors read and approved the final manuscript.

## Conflict of Interest Statement

The authors declare that the research was conducted in the absence of any commercial or financial relationships that could be construed as a potential conflict of interest.
